# Online Learning: A Panacea in the Time of COVID-19 Crisis

**DOI:** 10.1177/0047239520934018

**Published:** 2020-09

**Authors:** Shivangi Dhawan

**Affiliations:** 1 Department of Commerce, SGTB Khalsa College, University of Delhi, Delhi, India; 2 Department of Commerce, Delhi School of Economics, University of Delhi, Delhi, India

**Keywords:** coronavirus, COVID-19, education, online learning, technology, EdTech

## Abstract

Educational institutions (schools, colleges, and universities) in India are currently based only on traditional methods of learning, that is, they follow the traditional set up of face-to-face lectures in a classroom. Although many academic units have also started blended learning, still a lot of them are stuck with old procedures. The sudden outbreak of a deadly disease called Covid-19 caused by a Corona Virus (SARS-CoV-2) shook the entire world. The World Health Organization declared it as a *pandemic*. This situation challenged the education system across the world and forced educators to shift to an online mode of teaching overnight. Many academic institutions that were earlier reluctant to change their traditional pedagogical approach had no option but to shift entirely to online teaching–learning. The article includes the importance of online learning and Strengths, Weaknesses, Opportunities, & Challenges (SWOC) analysis of e-learning modes in the time of crisis. This article also put some light on the growth of EdTech Start-ups during the time of pandemic and natural disasters and includes suggestions for academic institutions of how to deal with challenges associated with online learning.

The deadly and infectious disease Corona Virus also known as Covid-19 has deeply affected the global economy. This tragedy has also shaken up the education sector, and this fear is likely to resonate across the education sector globally. The Covid-19 pandemic outbreak forced many schools and colleges to remain closed temporarily. Several areas are affected worldwide and there is a fear of losing this whole ongoing semester or even more in the coming future. Various schools, colleges, and universities have discontinued in-person teaching. As per the assessment of the researchers, it is uncertain to get back to normal teaching anytime soon. As social distancing is preeminent at this stage, this will have negative effects on learning opportunities. Educational units are struggling to find options to deal with this challenging situation. These circumstances make us realize that scenario planning is an urgent need for academic institutions (Rieley, 2020). This is a situation that demands humanity and unity. There is an urgent need to protect and save our students, faculty, academic staff, communities, societies, and the nation as a whole.

Several arguments are associated with e-learning. Accessibility, affordability, flexibility, learning pedagogy, life-long learning, and policy are some of the arguments related to online pedagogy. It is said that online mode of learning is easily accessible and can even reach to rural and remote areas. It is considered to be a relatively cheaper mode of education in terms of the lower cost of transportation, accommodation, and the overall cost of institution-based learning. Flexibility is another interesting aspect of online learning; a learner can schedule or plan their time for completion of courses available online. Combining face-to-face lectures with technology gives rise to blended learning and flipped classrooms; this type of learning environment can increase the learning potential of the students. Students can learn anytime and anywhere, thereby developing new skills in the process leading to life-long learning. The government also recognizes the increasing importance of online learning in this dynamic world.

The severe explosion of Corona Virus disease can make us add one more argument in terms of online learning, that is, online learning serves as a panacea in the time of crisis.

## Literature Review

### Online Learning or E-Learning

Rapid developments in technology have made distance education easy (McBrien et al., 2009). “Most of the terms (online learning, open learning, web-based learning, computer-mediated learning, blended learning, m-learning, for ex.) have in common the ability to use a computer connected to a network, that offers the possibility to learn from anywhere, anytime, in any rhythm, with any means” (Cojocariu et al., 2014).Online learning can be termed as a tool that can make the teaching–learning process more student-centered, more innovative, and even more flexible. Online learning is defined as “learning experiences in synchronous or asynchronous environments using different devices (e.g., mobile phones, laptops, etc.) with internet access. In these environments, students can be anywhere (independent) to learn and interact with instructors and other students” (Singh & Thurman, 2019). The synchronous learning environment is structured in the sense that students attend live lectures, there are real-time interactions between educators and learners, and there is a possibility of instant feedback, whereas asynchronous learning environments are not properly structured. In such a learning environment, learning content is not available in the form of live lectures or classes; it is available at different learning systems and forums. Instant feedback and immediate response are not possible under such an environment (Littlefield, 2018). Synchronous learning can provide a lot of opportunities for social interaction (McBrien et al., 2009). Amidst this deadly virus spread such online platforms are needed where (a) video conferencing with at least 40 to 50 students is possible, (b) discussions with students can be done to keep classes organic, (c) internet connections are good, (d) lectures are accessible in mobile phones also and not just laptops, (e) possibility of watching already recorded lectures, and (f) instant feedback from students can be achieved and assignments can be taken (Basilaia et al., 2020).

### Online Teaching Is No More an Option, It Is a Necessity

The major part of the world is on quarantine due to the serious outbreak of this global pandemic Covid-19 and therefore many cities have turned into phantom cities and its effects can be seen in schools, colleges, and universities too. Betwixt all this online teaching and online learning can be termed as the *panacea for the crisis*. The Corona Virus has made institutions to go from offline mode to online mode of pedagogy. This crisis will make the institutions, which were earlier reluctant to change, to accept modern technology. This catastrophe will show us the lucrative side of online teaching and learning. With the help of online teaching modes, we can sermonize a large number of students at any time and in any part of the world. All institutions must scramble different options of online pedagogical approaches and try to use technology more aptly. Many universities around the world have fully digitalized their operations understanding the dire need of this current situation. Online learning is emerging as a victor ludorum amidst this chaos. Therefore, the quality enhancement of online teaching–learning is crucial at this stage. Online education in Chinese universities has increased exponentially after the Covid-19 outbreak. There was an overnight shift of normal classrooms into e-classrooms, that is, educators have shifted their entire pedagogical approach to tackle new market conditions and adapt to the changing situations. During this tough time, the concern is not about whether online teaching–learning methods can provide quality education, it is rather how academic institutions will be able to adopt online learning in such a massive manner (Carey, 2020).

Resistance to change will not help any educational unit across the world. They will be judged on their pace to adapt to the changes in such a short period and their ability to maintain the quality. The reputation of educational units is on stake and under scrutiny. How well they behave and how well they maintain their quality of education amidst this crisis shows their adapting capabilities. The shift from face-to-face lectures to online classes is the only possible solution. Indeed, academic institutions would not be able to transform all of their college curricula into and online resource overnight. Distance, scale, and personalized teaching and learning are the three biggest challenges for online teaching. Innovative solutions by institutions can only help us deal with this pandemic (Liguori & Winkler, 2020). There is a requirement of a quick shift to online learning mode; therefore, the products by Google can be really useful under such problematic situations; they are (a) Gmail, (b) Google Forms, (c) Calendars, (d) G-Drive, (e) Google Hangouts, (f) Google Jam board and Drawings, (g) Google Classroom, and (h) Open Board Software (not a Google product, helps in recording meetings in the form of files). These tools can successfully be used as an alternative for face-to-face classes (Basilaia et al., 2020).

### Problems Associated With Online Teaching and Learning

There are *n* number of technologies available for online education but sometimes they create a lot of difficulties. These difficulties and problems associated with modern technology range from downloading errors, issues with installation, login problems, problems with audio and video, and so on. Sometimes student finds online teaching to be boring and unengaging. Online learning has so much of time and flexibility that students never find time to do it. Personal attention is also a huge issue facing online learning. Students want two-way interaction which sometimes gets difficult to implement. The learning process cannot reach its full potential until students practice what they learn. Sometimes, online content is all theoretical and does not let students practice and learn effectively. Mediocre course content is also a major issue. Students feel that lack of community, technical problems, and difficulties in understanding instructional goals are the major barriers for online learning (Song et al., 2004). In a study, students were found to be not sufficiently prepared for balancing their work, family, and social lives with their study lives in an online learning environment. Students were also found to be poorly prepared for several e-learning competencies and academic-type competencies. Also, there is a low-level preparedness among the students concerning the usage of Learning Management Systems (Parkes et al., 2014).

### Possible Solutions for Problems

A lot of issues are attached to online education but we cannot ignore the perks of it in times of such crisis. We can always have solutions to fix these difficulties. Technical difficulties can be solved through prerecording video lectures, testing the content, and always keeping Plan B ready so that the teaching–learning process cannot be hampered. Online courses should be made dynamic, interesting, and interactive. Teachers should set time limits and reminders for students to make them alert and attentive. Efforts should be made to humanize the learning process to the best extent possible. Personal attention should be provided to students so that they can easily adapt to this learning environment. Social media and various group forums can be used to communicate with students. Communication is the key when it gets difficult to try reaching out to students via texts, various messaging apps, video calls, and so on—content should be such that enable students for practice and also hone their skills. The quality of the courses should be improved continuously and teachers must try to give their best. Online programs should be designed in such a way that they are creative, interactive, relevant, student-centered, and group-based (Partlow & Gibbs, 2003). Educators must spend a lot of time in making effective strategies for giving online instructions. Effective online instructions facilitate feedback from learners, make learners ask questions, and broaden the learner horizon for the course content (Keeton, 2004). Institutions must focus on pedagogical issues and emphasize collaborative learning, case learning, and project-based learning through online instructions (Kim & Bonk, 2006).

The challenge to educational institutions is not only finding new technology and using it but also reimagining its education, thereby helping students and academic staff who are seeking guidance for digital literacy.

## Objectives of the Study

1. To explore the growth of EdTech Start-ups and online learning.
2. To conduct an Strengths, Weaknesses, Opportunities, & Challenges (SWOC) analysis of online learning during the Corona Virus pandemic and natural disasters.
3. To give some suggestions and recommendations for the success of online mode of learning during a crisis-like situation.

## Research Methodology

The study is descriptive and tries to understand the importance of online learning in the period of a crisis and pandemics such as the Covid-19. The problems associated with online learning and possible solutions were also identified based on previous studies. The SWOC analysis was conducted to understand various strengths, weaknesses, opportunities, and challenges associated with online mode of learning during this critical situation. The research tool used for analyzing the data which amassed from different sources for this study is a content analysis and the research method is descriptive research. We have taken into consideration the qualitative aspects of the research study. This study is completely based on the secondary data. A systematic review was done in detail for the collected literature.

Secondary sources of data used are (a) journals, (b) reports, (c) search engines, (d) company websites and scholarly articles, (e) research papers, and other academic publications.

## EdTech Start-ups in the Times of Corona

If we go back in history and see EdTech through the ages, we can observe that writing slates were used in Indian schools during the 1100s. In the year 1440, first printing press was invented by Johannes Guttenberg; in the 1600s, Abacus helped students in understanding fundamentals of Math; and in the year 1913, Thomas Edison promoted film clips as a replacement for teachers. In 1927, Sidney Pressy invented the first teaching machine famously called the MCQ machine. In the 1960s, online education originated at the University of Illinois and in 1994, India’s EdTech journey finally began in India with the launch of Educomp. Recently, around 2010, EdTechs start-ups entered the market intending to disrupt the education sector. A learning application Byju’s became one of the most valued EdTech companies in the year 2019. And from then many start-ups have come up to give tough competition to Byjus’s. Li Kang, Ai English Executive Director said, “Online Learning is the future and if there was no virus, that realization would have taken another few years but this has accelerated the process.”

EdTech Start-ups are tapping all the right opportunities by providing free online courses to students amidst this crisis. UNESCO also suggested that these EdTech Start-ups and learning apps can help students during such hard times. Digital payment companies, such as Paytm, Mobiwik, Tez, PhonePe, and so on, grew rapidly during and after demonetization. Now, in this pandemic outbreak, EdTech start-ups are hoping for improved performance. EdTech start-ups are trying hard to make most out of this situation by providing several free courses and e-resources to the students. Although the availability of electricity and a stable internet connection is still a bigger challenge in their way as a lot of Indian cities especially small cities still face frequent electricity shortages. As per the reports, initiatives by these companies are already bringing them gains. Their customer base is improving a lot, it might be for a temporary period but even if they can retain a few customers it is for their good only.

Educators or teachers in the form of facilitators face a lot of trouble while working on these EdTech start-ups in the form of how to start using it when to use it, how to reduce distractions for students, how to hone students’ skills via EdTech. The participation by students is not enough, educators must put considerable effort to increase student engagement, retain their attention, take feedbacks, and assess them in several ways. This will create an effective and meaningful learning environment. EdTech cannot replace a teacher but it can enhance instruction. During such tough times, when Covid-19 has forced schools and colleges to remain completely lockdown for few weeks due to the seriousness of the situation, EdTech companies can prove to be of great help to students (Brianna et al., 2019). According to the reports by KPMG and Google, the EdTech sector will boom and is likely to reach around 2 Billion Dollars by 2021. Some of the famous EdTech start-ups include Byju’s, Adda247, Alolearning, AptusLearn, Asmakam, Board Infinity, ClassPlus, CyberVie, Egnify, Embibe, ExtraaEdge, iStar, Jungroo Learning, GlobalGyan, Lido Learning, Pesto, Vedantu, Edubrisk, ZOOM Classroom, ZOOM Business, Toppr, Unacademy, Coursera, Kahoot, Seesaw, Khan Academy, e-pathshala, GuruQ, and the list is long. SWAYAM portal is an interesting educational program that is initiated by the government of India to achieve three important objectives of our educational policy, that is, access, equity, and quality. The main objective of SWAYAM is to provide online learning and reduce the digital divide. It provides a large number of free courses for school, distance, graduate, and postgraduate education. During the Covid-19 crisis, SWAYAM is of great help for students across the country.

## SWOC Analysis of Online Learning: During Corona Virus Pandemic and Other Crisis-Like Situation (Natural Disasters)

In the aftermath of some of the natural calamities such as floods, cyclones, earthquakes, hurricanes, and so on, knowledge delivery becomes a challenging task. These hazards disrupt the educational processes in schools and colleges in several ways. Sometimes, it leads to closure of schools and colleges which creates serious consequences for students and deprives them of their fundamental right to education and poses them to future risk. “100 million children and young people are affected by natural disasters every year. Most of them face disruption to their schooling” (World Vision). Situations of crisis and conflicts are the biggest hurdles in the path of education. Many students and teachers also face psychological problems during crisis—there is stress, fear, anxiety, depression, and insomnia that lead to a lack of focus and concentration. Disasters create havoc in the lives of people (Di Pietro, 2017).

With changing weather patterns and rising global temperatures, an increasing number of extreme weather events have become the new norm. Such events caused varying amounts of loss to life and property. Table1 shows some of the natural disasters that caused huge disruption in educational processes. Large numbers of schools and colleges were destroyed and thousands of students were affected by these natural calamities. Their education got disrupted in midway. “Disruption of education can leave children at risk of child labor, early marriage, exploitation, and recruitment into armed forces” (Baytiyeh, 2018). When disasters and crises (man-made and natural) occur, schools and colleges need to be resilient and should find new ways to continue with teaching–learning activities (Chang-Richards et al., 2013).

For instance, in 2016, Italy experienced three violent and powerful earthquakes. This brought huge devastation in the number of areas. About 1,00,000 people became homeless, buildings and structures collapsed, and there was severe loss of life and property. The University of Camerino, one of the oldest universities in the world suffered a huge loss. The university was in crisis, its structure collapsed, a large number of students became homeless and some left the place. In such situations, students were deprived of education and learning. It is rightly said, “It is difficult to stick to the traditional road when the road itself has crumbled.” This means that face-to-face instructions were not possible at that time; therefore, management and leaders came forward to devise some plans to keep the educational processes in continuation. Before the earthquake’s destruction, e-learning at the University was cumbersome. But they were unstoppable, and to continue the teaching–learning processes, they used Webex (an online tool) by Cisco. Webex helped professors in designing their instructional programs and sharing notes and presentations with students. In almost 1 month, the university was well-versed with e-learning strategies and techniques. They integrated themselves well in an e-learning world. They believed that, of course, the value of the face-to-face instruction method cannot be reduced, but e-learning can be used together with the traditional methods to bring in efficiency, effectiveness, and competitive edge over other competitors by imparting quality education (Barboni, 2019).

In February 2011, a 6.3 magnitude earthquake shook Christchurch and the University of Canterbury collapsed. Information technology and online learning helped the university to restart its operations and gave them a second life (Todorova & Bjorn-Andersen, 2011).

At New Orleans, Southern University converted itself into an e-learning campus after the violent hurricane created a Havoc. Several online courses were offered and mobiles were used to provide education to the displaced students (Omar et al., 2008).

**Figure 1.**
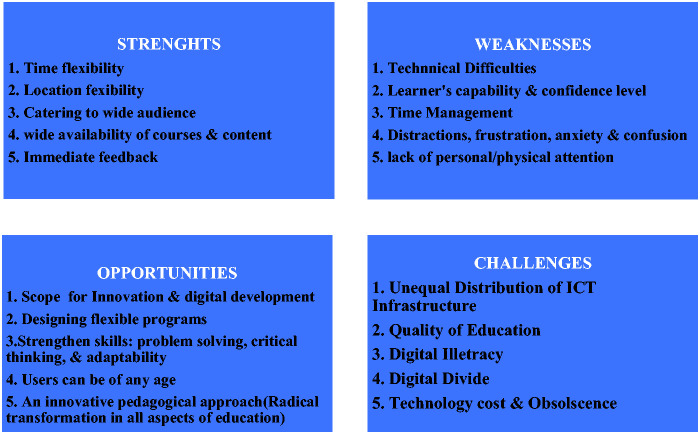
The SWOC Analysis of Online Learning During Such Crises.*Note*. SWOC = Strengths, Weaknesses, Opportunities, & Challenges.

And the most recent disaster is in the form of the Covid-19 which is spreading like a forest fire around the world. All of the schools, colleges, and universities are facing lockdowns in the most affected areas to curb further spread of the Corona Virus. Many academic institutions are, therefore, seeking the help of online learning so that teaching and learning processes are not hampered. The SWOC Analysis of Online Learning is shown in Figure 1.

In the last few years, e-learning has started gaining popularity in India. Many platforms provide affordable courses to students via Massive Open Online Courses. Still a lot of institutions in India were reluctant toward online teaching and learning. However, the challenges posed by the Corona Virus pandemic introduced everyone to a new world of online learning and remote teaching. Instructors indulged them in remote teaching via few flatforms such as Google Hangouts, Skype, Adobe Connect, Microsoft teams, and few more, though ZOOM emerged as a clear winner. Also, to conduct smooth teaching–learning programs, a list of online etiquettes was shared with students and proper instructions for attending classes were given to them (Saxena, 2020).

**Table 1.** Natural Disaster That Affected Teaching–Learning Badly.

Year	Natural disasters
2009	A violent earthquake in 9 the city of L’Aquila
2010	Floods in Pakistan
2011	Tropical storm Washi in the Philippines
2011	A series of earthquakes in New Zealand
2013	Tropical storm Haiyan in the Philippines
2015	Gorkha floods in Nepal
2017	Harvey and Irma Hurricanes in the United States
2017	Floods in Nepal, Bangladesh, and India
2018	An earthquake in Papua New Guinea
2018	Earthquakes and tsunamis in Indonesia
2019	The typhoon Lekima in China
2019	The typhoon Hagibis in Japan
2019	The tropical cyclone Idai in Southeastern Africa
2019	The heat wave in Bihar

*Source.* Save the Children (2014, 2017), US News and World Report, & Briggs, 2018.

### Strengths

E-learning methods and processes are really strong. These strengths of the online learning modes can rescue us from these hard times. It is student-centered and offers a great deal of flexibility in terms of time and location. The e-learning methods enable us to customize our procedures and processes based on the needs of the learners. There are plenty of online tools available which is important for an effective and efficient learning environment. Educators can use a combo of audio, videos, and text to reach out to their students in this time of crisis to maintain a human touch to their lectures. This can help in creating a collaborative and interactive learning environment where students can give their immediate feedback, ask queries, and learn interestingly. The *Anywhere-Anytime* feature of e-learning is beneficial in the times of crisis-like situation, for instance, man-made disasters, natural disasters, or pandemics such as Covid-19. The closure of places and unsafe traveling by roads can create a lot of troubles but e-learning will at least not keep us deprived of getting an education at our homes or workplaces.

Technology provides innovative and resilient solutions at times of crisis to combat disruption and helps people to communicate and even work virtually without the need of face-to-face interaction. This leads to many system changes in organizations as they adopt new technology for interacting and working (Mark & Semaan, 2008).

### Weaknesses

E-learning has certain weaknesses in the form that it can hamper the communication between the learner and the educator, that is, direct communication and human touch are lost. Users can face many technical difficulties that hinder and slow-down the teaching–learning process (Favale et al., 2020). Time and location flexibility, though it is the strength of online learning these aspects are fragile and create problems. Student’s nonserious behavior in terms of time and flexibility can cause a lot of problems. All students and learners are not the same, they vary in degrees of their capabilities and confidence level. Some do not feel comfortable while learning online, leading to increased frustration and confusion. Inadequate compatibility between the design of the technology and component of psychology required by the learning process; and inadequate customization of learning processes can obstruct the teaching process and creates an imbalance.

### Opportunities

Online learning generally has a lot of opportunities available but this time of crisis will allow online learning to boom as most academic institutions have switched to this model. Online Learning, Remote Working, and e-collaborations exploded during the outbreak of Corona Virus crisis (Favale et al., 2020). Now, academic institutions can grab this opportunity by making their teachers teach and students learn via online methodology. The people have always been complacent and never tried some new modes of learning. This crisis will be a new phase for online learning and will allow people to look at the fruitful side of e-learning technologies. This is the time when there is a lot of scope in bringing out surprising innovations and digital developments. Already, EdTech companies are doing their bit by helping us fighting the pandemic and not letting learning to be put at a halt. Teachers can practice technology and can design various flexible programs for students’ better understanding. The usage of online learning will test both the educator and learners. It will enhance problem-solving skills, critical thinking abilities, and adaptability among the students. In this critical situation, users of any age can access the online tools and reap the benefits of time and location flexibility associated with online learning. Teachers can develop innovative pedagogical approaches in this panicky situation, now also termed as *Panic*gogy. EdTech Start-ups have plenty of opportunities to bring about radical transformations in nearly all the aspects associated with education ranging from, teaching, learning, evaluation, assessment, results, certification, degrees, and so on. Also, increasing market demand for e-learning is an amazing opportunity for EdTech start-ups to bring technological disruption in the education sector.

### Challenges

Online learning faces many challenges ranging from learners’ issues, educators’ issues, and content issues. It is a challenge for institutions to engage students and make them participate in the teaching–learning process. It is a challenge for teachers to move from offline mode to online mode, changing their teaching methodologies, and managing their time. It is challenging to develop content which not only covers the curriculum but also engage the students (Kebritchi et al., 2017). The quality of e-learning programs is a real challenge. There is no clear stipulation by the government in their educational policies about e-learning programs. There is a lack of standards for quality, quality control, development of e-resources, and e-content delivery. This problem needs to be tackled immediately so that everyone can enjoy the benefits of quality education via e-learning (Cojocariu et al., 2014). One should not merely focus on the pros attached to the adoption of online learning during the crises but should also take account of developing and enhancing the quality of virtual courses delivered in such emergencies (Affouneh et al., 2020). A lot of time and cost is involved in e-learning. It is not as easy as it seems, a considerable amount of investment is needed for getting the devices and equipment, maintaining the equipment, training the human resources, and developing the online content. Therefore, an effective and efficient educational system needs to be developed to impart education via online mode.

Ensuring digital equity is crucial in this tough time. Not all the teachers and students have access to all digital devices, internet, and Wi-Fi. Unavailability of proper digital tools, no internet connections, or iffy Wi-Fi connections can cause a lot of trouble due to which many students might lose out learning opportunities. Efforts should be taken by institutions to ensure that every student and faculty is having access to the required resources. They must also ensure that all the educational apps work on mobile phones as well, in case students do not have laptops. Therefore, steps must be taken to reduce the digital divide.

*Practice makes a man perfect* is a famous and very true proverb. Students and teachers across various universities have never really practiced e-learning. Most of them are complacent and are stuck with traditional modes of teaching. The Corona Virus outbreak is the chance to make out the best from the current situation. We can learn a lot in this challenging situation. A lot of tools are available, teachers are required to choose the best tool and implement it to impart education to their students. A step-by-step guide can be prepared by academic institutions that can guide the teachers and students on how to access and use various e-learning tools and how to cover major curriculum content via these technologies thereby reducing the digital illiteracy. Teachers can present the curriculum in various formats, that is, they can use videos, audios, and texts. It is beneficial if educators complement their lectures with video chats, virtual meetings, and so on to get immediate feedback and maintain a personal connection with the students.

## Conclusions and Suggestions

Ayebi-Arthur (2017) conducted a case study of a college in New Zealand which was badly affected by seismic activities. In her study, she found that the college became more resilient to online learning after that disastrous event. Technology helped them overcome the barriers in those difficult times. But they suggest that robust IT Infrastructure is a prerequisite for online learning. Infrastructure needs to be so strong that it can provide unhindered services during and after the crisis.

As per the World Economic Forum, the Covid-19 pandemic also has changed the way how several people receive and impart education. To find new solutions for our problems, we might bring in some much-needed innovations and change. Teachers have become habitual to traditional methods of teaching in the form of face-to-face lectures, and therefore, they hesitate in accepting any change. But amidst this crisis, we have no other alternative left other than adapting to the dynamic situation and accepting the change. It will be beneficial for the education sector and could bring a lot of surprising innovations. We cannot ignore and forget the students who do not have access to all online technology. These students are less affluent and belong to less tech-savvy families with financial resources restrictions; therefore, they may lose out when classes occur online. They may lose out because of the heavy costs associated with digital devices and internet data plans. This digital divide may widen the gaps of inequality.

This terrible time of fate has taught us that everything is unpredictable and we need to be ready to face challenges. Although this outbreak did not give us much time to plan we should take a lesson from this that planning is the key. We should plan everything, no matter if plan A fails, we should have plan B ready. This can only be done if we do scenario planning. There is a need to prioritize all the critical and challenging situations which may occur and plan accordingly. This pandemic has also taught us that students must possess certain skills such as skills of problem-solving, critical thinking, and most importantly adaptability to survive the crisis. Educational institutions must build resilience in their systems to ensure and prioritize the presence of these skills in their students.

“The key lesson for others may be to embrace e-learning technology before disaster strikes!” (Todorova & Bjorn-Andersen, 2011). Today, we are forced to practice online learning, things would have been different if we have already mastered it. The time we lost in learning the modes could have been spent on creating more content. But it is better late than never. This virus surely has accelerated the process of online learning. For instance, this e-application called ZOOM is making a lot of news because of its viable features. It allows conducting live online classes, web-conferencing, webinars, video chats, and live meetings. As most of the schools, colleges, universities, companies are closed due to lockdowns/curfews and most of the people are working from home, this app helped in keeping people connected via video conferencing. This application is trending on Google play store amidst the ongoing crisis. People are practicing social distancing so this application gave them a sigh of relief. ZOOM also allows conducting business meetings.

Disasters will continue to occur and technologies will likely help us cope with them (Meyer & Wilson, 2011). Don Dippo, The Co-Principal Investigator at the Borderless Higher education for Refugees said that “We are in a world where conflict and environmental destruction … are going to have lots of people, families, and communities, living in precarious contexts. The willingness of post-secondary institutions to step-up and engage and provide opportunities for those people will never be as large as the need. The only way we can even make a dent in this is to learn to collaborate and cooperate across institutions and across time and spatial boundaries. The only way really to do that is to rely on technology to create conditions to allow people to collaborate.”

We need a high level of preparedness so that we can quickly adapt to the changes in the environment and can adjust ourselves to different delivery modes, for instance, remote learning or online learning in situations of pandemics such as Covid-19. Institutions and organizations should prepare contingency plans to deal with challenges such as pandemics and natural disasters (Seville et al., 2012). Reliability and sufficient availability of Information Communication Technology infrastructure, learning tools, digital learning resources in the form of Massive Open Online Courses, e-books, e-notes, and so on are of utmost importance in such severe situations (Huang et al., 2020). Instruction, content, motivation, relationships, and mental health are the five important things that an educator must keep in mind while imparting online education (Martin, 2020). Some teaching strategies (lectures, case-study, debates, discussions, experiential learning, brainstorming sessions, games, drills, etc.) can be used online to facilitate effective and efficient teaching and learning practices. In such panicky situations, where the lives of so many people are at stake, teaching and learning should be made interesting. This will also reduce the stress, fear, and anxiety levels of people. For this, proper technique and learning support should be provided to teachers and students and government support is also crucial at such stage. Pedagogical and technical competency of online educators is of utmost importance. Rigorous quality management programs and continuous improvement are pivotal for online learning success and making people ready for any crisis-like situation.

Natural disasters can stimulate our motivation for the adoption of highly innovative communication technology and e-learning tools (Tull et al., 2017). To make e-learning effective in such difficult times, we need to focus on the use of technology more efficiently, that is, the usage of that technology which has minimum procurement and maintenance costs but can effectively facilitate educational processes. Before bringing in and adopting any e-learning tool or technology, its pros and cons need to be weighed. Institutions should conduct plenty of research when bringing the right technology for different educational initiatives. There should be proper clarity on the purpose and context of technology adoption. As several factors affect the choice of a particular technology such as security features, availability and condition of laboratories, internet speed, internet access, digital literacy levels of the beneficiaries, and so on. E-learning can help in providing inclusive education even at the time of crisis. Such systems need to be developed in educational institutions that make sure that no student is getting deprived of education due to their location, social class, ethnicity, and so on. Online methods of teaching support and facilitate learning–teaching activities, but there is a dire need to weigh the pros and cons of technology and harness its potentials. Disasters and pandemic such as Covid-19 can create a lot of chaos and tensions; therefore, there is an important need to study the technology deeply and with due diligence to balance these fears and tensions amidst such crisis.

## Authors’ Note

Below are the sites of different educational portals which I referred to while knowing what types of courses they offer & two of them are news articles as COVID is currently in the news. https://www.ft.com/ https://swayam.gov.in/explorer https://bolog.zoom.us/ https://byjus.com/ https://economictimes.indiatimes.com/small-biz/startups/features/covid-19-cities-lockdown-zoom-video-calling-app-videoconferencing-google-hangout-skype/articleshow/74767206.cms https://www.usatoday.com https://www.wvi.org/education-and-life-skills/education-emergencies (World Vision)
